# A comparison of diagnostic methods for canine Ehrlichiosis: Microscopy and RNases hybridization-assisted amplification technology compared with the quantitative polymerase chain reaction

**DOI:** 10.14202/vetworld.2025.1214-1223

**Published:** 2025-05-17

**Authors:** Wisut Prasitsuwan, Thanikran Suwannachote, Thirawat Sumalai, Rachakris Lertpatarakomol, Tassanee Trairatapiwan, Sakchai Ruenphet

**Affiliations:** 1 Animal Biotechnology, Mahanakorn University of Technology, Nong Chock, Bangkok, 10530, Thailand; 2 Clinic for Small Domestic Animals and Radiology, Mahanakorn University of Technology, Nong Chock, Bangkok, 10530, Thailand

**Keywords:** canine blood parasites, ehrlichiosis, microscopy, quantitative polymerase chain reaction, RNase hybridization-assisted amplification test kit

## Abstract

**Background and Aim:**

Canine vector-borne pathogens, particularly blood parasites, pose significant health threats to domestic dogs, ranging from subclinical infections to severe systemic diseases. In Thailand, microscopic examination remains the standard diagnostic method, despite its limitations. This study aimed to (i) determine the prevalence of major canine vector-borne pathogens in Bangkok, Thailand during the 2024 rainy season and (ii) evaluate the diagnostic performance of microscopy and a novel RNase hybridization-assisted amplification (RHAM) test kit in detecting canine Ehrlichiosis, compared to the quantitative polymerase chain reaction (qPCR) gold standard.

**Materials and Methods:**

A total of 134 whole blood samples were collected from clinically suspected dogs. Microscopy was performed on Giemsa-stained blood smears, and the RHAM test kit was employed for nucleic acid detection. qPCR served as the reference method. Sensitivity, specificity, accuracy, and precision of each diagnostic method were calculated relative to qPCR results.

**Results:**

Microscopic examination revealed the following infection prevalences: *Ehrlichia* spp. (26.12%), *Babesia* spp. (4.48%), *Hepatozoon canis* (6.72%), *Anaplasma* spp. (0.75%), *Dirofilaria immitis* (3.73%), and *Brugia* spp. (3.73%). Compared with qPCR, microscopy demonstrated a sensitivity of 51.47%, specificity of 87.88%, accuracy of 69.40%, and precision of 81.39% for Ehrlichiosis detection. In contrast, the RHAM test kit achieved markedly higher diagnostic metrics: Sensitivity (91.18%), specificity (98.48%), accuracy (94.78%), and precision (98.41%). Notably, the RHAM kit provided rapid, user-friendly detection, approximating qPCR diagnostic performance, although its sensitivity slightly declined in samples with very low pathogen titers.

**Conclusion:**

This study highlights the continued high prevalence of Ehrlichiosis among dogs in Bangkok during the rainy season. Although microscopy remains practical, its diagnostic limitations are significant. The RHAM test kit demonstrated excellent sensitivity and specificity, offering a rapid and accurate alternative for Ehrlichiosis detection, particularly suitable for resource-limited settings lacking qPCR capabilities. Adoption of the RHAM assay could improve early diagnosis and management of canine Ehrlichiosis at grassroots veterinary facilities.

## INTRODUCTION

Ticks are obligate hematophagous ectoparasites that serve as vectors for a wide array of pathogens, including viruses, bacteria, rickettsiae, and protozoa, thereby playing a critical role in the global dissemination of animal diseases [1]. Among these, *Rhipicephalus sanguineous* is the predominant tick species parasitizing domestic dogs throughout Southeast Asia, including Cambodia, Laos, Peninsular Malaysia, Myanmar, Vietnam, and Thailand [2]. Dense domestic dog populations provide an ideal environment for *R. sanguineus* proliferation, thereby enhancing the risk of tick-borne disease transmission. Pathogens such as *Anaplasma* spp., *Ehrlichia* spp., and *Rickettsia* spp. transmitted by ticks are increasingly recognized as important zoonotic agents, posing substantial public health concerns for companion animals and humans alike. In particular, *Babesia* spp., *Ehrlichia canis*, *Hepatozoon* spp., *Anaplasma platys*, and *Mycoplasma* spp. are among the most frequently identified vector-borne pathogens affecting canine populations in Southeast Asia, contributing markedly to elevated morbidity and mortality rates [3–7]. Furthermore, *Dirofilaria immitis* and *Brugia* spp. are widespread vector-borne parasites of dogs, cats, and humans, distributed across tropical, subtropical, and certain temperate regions. *D. immitis*, the principal etiological agent of canine and feline heartworm disease, is characterized by the subperiodic circulation of microfilariae in the peripheral blood without clear diurnal or nocturnal peaks. Beyond veterinary significance, *D. immitis* also causes pulmonary dirofilariasis in humans, highlighting its broader zoonotic potential. Similarly, lymphatic filariasis, attributed to filarial nematodes of the genus *Brugia*, remains a neglected tropical disease affecting approximately 80 countries, impacting both human and animal populations [8].

Canine blood parasites induce a broad spectrum of clinical manifestations, ranging from asymptomatic infections to severe systemic diseases involving hematological and multiple organ dysfunction [9, 10]. Common clinical signs include fever, hemolytic anemia, thrombocytopenia, splenomegaly, and multi-organ impairment [10, 11]. Coinfections involving multiple blood parasites significantly complicate diagnostic processes, exacerbate clinical severity, diminish therapeutic efficacy, and adversely affect prognostic outcomes [12]. In Thailand, diagnosis of canine blood parasite infections predominantly relies on microscopic evaluation of Giemsa-stained blood smears. However, this conventional approach is limited by low sensitivity, especially in cases of low parasitemia, and requires considerable expertise for accurate interpretation [13]. Although serological assays are available for detecting *Ehrlichia* spp. and *Anaplasma* spp., these methods are unable to differentiate between past exposure and active infection, thus representing a critical diagnostic limitation [14]. Polymerase chain reaction (PCR) techniques have emerged as the preferred modality for confirmation of blood parasite infections, due to their superior sensitivity and specificity [13, 15–17]. Earlier molecular diagnostic efforts predominantly targeted the genus-specific disulfide bond formation protein (*dsb*) gene [18]. In the case of *E. canis*, species-specific PCR assays focusing on the *p30* and 16S ribosomal RNA (16S *rRNA*) genes have been developed, with the *p30* gene demonstrating greater specificity compared to 16S rRNA in nested PCR protocols [19].

Despite the widespread recognition of vector-borne diseases as major causes of morbidity and mortality in canine populations, diagnostic limitations persist, particularly in resource-constrained settings. Conventional microscopy, although widely utilized in Thailand and other Southeast Asian countries, demonstrates suboptimal sensitivity, especially in cases of low parasitemia, and relies heavily on examiner expertise. Serological methods, while useful, are unable to differentiate between active and past infections, thereby limiting their diagnostic precision. Although PCR-based diagnostics offer superior sensitivity and specificity, their cost, technical complexity, and requirement for specialized laboratory infrastructure restrict their broader implementation in clinical veterinary practice. Recent advances, such as RNase hybridization-assisted amplification (RHAM) technology, have introduced novel nucleic acid detection platforms that promise rapid, accurate, and field-deployable diagnostics; however, robust field validation of RHAM technology in naturally infected canine populations remains limited. Moreover, comprehensive studies comparing the diagnostic performance of RHAM against both traditional microscopy and gold-standard qPCR assays for detecting *Ehrlichia* spp. under endemic field conditions are currently lacking.

The present study was designed to address these diagnostic challenges by systematically evaluating the prevalence of major canine vector-borne pathogens during the 2024 rainy season in Bangkok, Thailand, and by comparatively assessing the diagnostic performance of microscopy, the RHAM Ehrlichiosis test kit, and quantitative PCR (qPCR) for detecting canine Ehrlichiosis. Specifically, this investigation aimed to (i) determine the prevalence rates of *Ehrlichia* spp., *Babesia* spp., *Hepatozoon canis*, *Anaplasma* spp., *D. immitis*, and *Brugia* spp. among clinically suspected dogs; (ii) evaluate the sensitivity, specificity, accuracy, and precision of microscopy and RHAM testing relative to qPCR results; and (iii) validate the field applicability and diagnostic robustness of the RHAM assay as a potential alternative for decentralized or resource-limited veterinary settings.

## MATERIALS AND METHODS

### Ethical approval and informed consent

The procedures involving animals were reviewed and approved by the Animal Research Ethics Committee of the Faculty of Veterinary Medicine, Mahanakorn University of Technology, Thailand (approval number: ACUC-MUT-2024/006). Informed consent was obtained from all dog owners, who signed an official document before sample collection, thereby ensuring adherence to ethical standards.

### Study period and location

Sample collection was conducted from June to August 2024, corresponding to the rainy season in Thailand, a period associated with a high prevalence of brown dog ticks. Blood samples were obtained from three veterinary facilities located on the outskirts of Bangkok: The Small Animal Teaching Hospital, Faculty of Veterinary Medicine, Mahanakorn University of Technology; Kling Kaew Animal Hospital; and Vet Home Polyclinic.

### Sample collection

A total of 134 whole blood samples were collected by a veterinarian from dogs of various breeds and ages presenting with clinical signs suggestive of blood parasite infection, such as fever, lethargy, emaciation, or pale mucous membranes. Blood samples were stored in ethylenediaminetetraacetic acid tubes (Accuette^®^, Greiner Bio-One Thailand, Chonburi, Thailand) at 4°C, and blood smears were prepared within 24 h post-collection. Residual samples were stored at −80°C until subsequent analysis by RHAM testing and quantitative polymerase chain reaction (qPCR) within 1 month.

### Microscopic examination

Microscopy was employed to detect vector-borne pathogens, including *Ehrlichia* spp., *Babesia* spp., *H. canis*, *Anaplasma* spp., *D. immitis*, and *Brugia* spp. Blood smears were prepared immediately after sample collection by placing a drop of blood near one end of a clean glass slide and using a second slide held at a 45° angle to spread the blood into a thin film. Following air-drying, smears were fixed in methanol and stained with 5% Giemsa solution for 20 min. Stained slides were rinsed with tap water, air-dried, and examined under a 100× oil immersion objective lens. Well-stained, populated fields were systematically examined across at least 100 fields of view. Experienced technologists, blinded to the qPCR and RHAM results, meticulously analyzed the Giemsa-stained smears to minimize observer bias.

### RHAM testing

**Figure 1:**
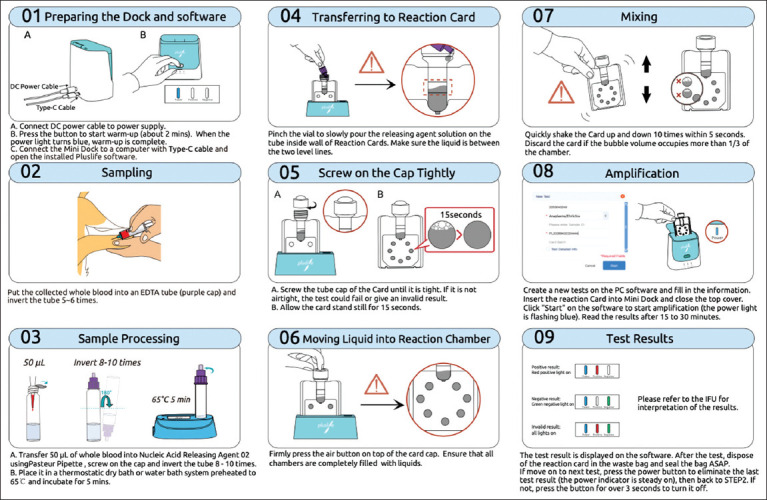
The operation process illustration of the RNase hybridization-assisted amplification test kit for the nucleic acid detection of *Ehrlichiosis*.

The RHAM test kit for Ehrlichiosis detection (Pluslife Integrated Nucleic Acid Testing Device, Guangzhou Pluslife Biotech, China) comprises three components: The nucleic acid testing device, a nucleic acid releaser tube, and the Pluslife *Anaplasma/Ehrlichi*a Nucleic acid test card. The detection procedure commenced with preheating the device, which was connected to the Pluslife Pet Application on a mobile device or computer. A 50 μL aliquot of whole blood was transferred into the nucleic acid releaser tube using a sterile pipette and incubated at 65°C for 5 min. The lysate was then dispensed into the designated injection line of the test card. After brief stabilization (15 s) on the Pluslife stand and air button activation, the card was gently shaken and inserted into the device. Amplification and detection were performed automatically, and results were read after 30 min. A detailed visual schematic of the RHAM operational workflow is described in Figure 1.

### qPCR

Genomic DNA extraction from blood samples, including positive and negative controls, was performed using the TANBead^®^ automated nucleic acid extraction system (Taiwan Advanced Nanotech, Taoyuan, Taiwan; Lot number E113021B) following the manufacturer’s protocol. Extraction was conducted using the Smart LabAssist -E13200 Automated Nucleic Acid Extractor (Taiwan Advanced Nanotech, Taiwan). Detection of the *E. canis* genome was performed using the Primerdesign^™^ geneSig^®^ kit (Primerdesign Ltd., UK) on a C 1,000 Touch Thermal Cycler (Bio-Rad Laboratories, California, USA). The qPCR thermal profile included an initial activation at 95°C for 2 min, followed by 50 cycles comprising denaturation at 95°C for 10 s and annealing/extension at 60°C for 60 s. Cycle threshold (Ct) values were used for result interpretation. A valid run required successful amplification of the positive control and no amplification in the negative control; otherwise, the assay was repeated.

### Statistical analysis

The apparent prevalence of *Ehrlichia* spp., *Babesia* spp., *H. canis*, *Anaplasma* spp., *D. immitis*, and *Brugia* spp. was calculated and expressed as percentages with corresponding 95% confidence intervals. Diagnostic performance metrics – including sensitivity, specificity, accuracy, and precision – were assessed by comparing microscopy and RHAM results against qPCR results, designated as the reference standard. Samples yielding results concordant with qPCR were classified as true positives or true negatives, while discordant samples were categorized as false positives or false negatives. Sensitivity, specificity, accuracy, and precision were subsequently calculated based on these classifications following standard formulas [20].


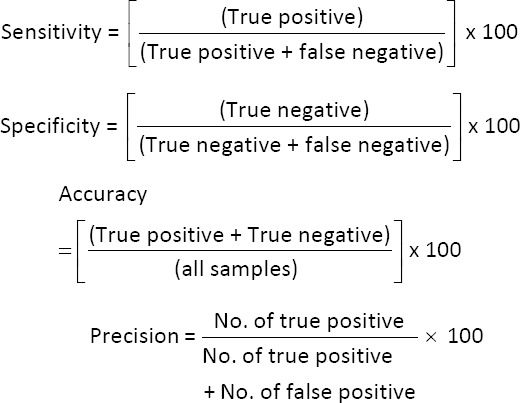


## RESULTS

### Prevalence of vector-borne pathogens by microscopy

**Figure 2:**
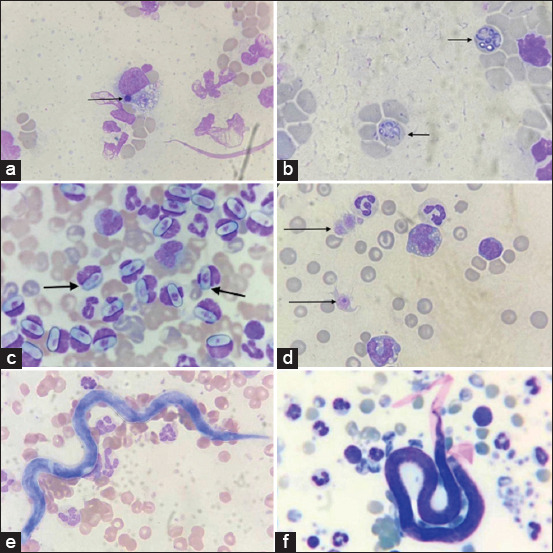
Blood pathogens in stained blood smear. (a) *Ehrlichia* spp. in monocytes, (b) *Babesia* spp. in red blood cells, (c) *Hepatozoon canis* in neutrophils, (d) *Anaplasma* spp. in platelets, (e) *Dirofilaria immitis*, and (f) *Brugia* spp.

The prevalence of vector-borne pathogens detected in 134 canine whole blood samples collected in Bangkok, Thailand during the 2024 rainy season is summarized in Figure 2 and Table 1. The overall prevalence rates were as follows: *Ehrlichia* spp. (26.12%), *Babesia* spp. (4.48%), *H. canis* (6.72%), *Anaplasma* spp. (0.75%), *D. immitis* (3.73%), and *Brugia* spp. (3.73%).

**Table 1:** Apparent prevalence of *Ehrlichia* spp., *Babesia* spp., *Hepatozoon* canis, *Anaplasma* spp., *Dirofilaria immitis*, and *Brugia* spp. using a microscope in 134 whole blood dog samples examined in Nong Chock and Ladkrabang, Bangkok, Thailand during the rainy season of 2024.

Pathogens	n	Percentage	Confidence interval (95%)
Overall			
*Ehrlichia* spp.	35	26.12	18.92–34.41
*Babesia* spp.	6	4.48	1.66–9.49
*Hepatozoon* canis	9	6.72	3.12–12.37
*Anaplasma* spp.	1	0.75	0.02–4.09
*Dirofilaria immitis*	5	3.73	1.22–8.49
*Brugia* spp.	5	3.73	1.22–8.49
Single infection			
*Ehrlichia* spp.	30	22.38	15.64–30.39
*Babesia* spp.	3	2.24	0.46–6.40
*Hepatozoon* canis	7	5.22	2.13–10.47
*Anaplasma* spp.	1	0.75	0.02–4.09
*Dirofilaria immitis*	2	1.49	0.18–5.29
*Brugia* spp.	1	0.75	0.02–4.09
Double infection			
*Ehrlichia* spp. + *Babesia* spp.	2	1.49	0.18–5.29
*Ehrlichia* spp. + *Hepatozoon* canis	1	0.75	0.02–4.09
*Ehrlichia* spp. + *Brugia* spp.	1	0.75	0.02–4.09
*Dirofilaria immitis*+*Brugia* spp.	3	2.24	0.46–6.40
Triple infection			
*Ehrlichia* spp. + *Babesia* spp. + *Hepatozoon* canis	1	0.75	0.02–4.09

Analysis of single infections revealed that *Ehrlichia* spp. was the most prevalent pathogen (22.38%), followed by *H. canis* (5.22%), *Babesia* spp. (2.24%), *D. immitis* (1.49%), *Anaplasma* spp. (0.75%), and *Brugia* spp. (0.75%).

Double infections were identified in several samples, including co-infections of *Ehrlichia* spp. and *Babesia* spp. (1.49%), *Ehrlichia* spp. and *H. canis* (0.75%), *Ehrlichia* spp. and *Brugia* spp. (0.75%), and *D. immitis* and *Brugia* spp. (2.24%).

Triple infections were observed in 0.75% of the samples, specifically involving *Ehrlichia* spp., *Babesia* spp., and *H. canis*.

### Comparison of microscopy and qPCR for *Ehrlichia* spp. detection

The diagnostic performance of microscopic examination for the detection of *Ehrlichia* spp. was evaluated against qPCR results, as detailed in Table 2. The performance metrics derived from this comparison (Table 3) demonstrated a sensitivity of 51.47%, specificity of 87.88%, accuracy of 69.40%, and precision of 81.39%.

**Table 2:** The results of microscopy for *Ehrlichia* spp. detection compared with qPCR in 134 whole blood dog samples examined in Nong Chock and Ladkrabang, Bangkok, Thailand.

No.	Microscope	qPCR		No.	Microscope	qPCR		No.	Microscope	qPCR		No.	Microscope	qPCR	
							
	Result	Result	Ct		Result	Result	Ct		Result	Result	Ct		Result	Result	Ct
1	Negative	Positive	32.57	41	Negative	Negative	-	81	Positive	Positive	29.83	121	Positive	Positive	33.30
2	Positive	Positive	23.49	42	Negative	Negative	-	82	Positive	Positive	30.24	122	Positive	Positive	31.99
3	Positive	Negative	-	43	Positive	Negative	-	83	Positive	Positive	29.96	123	Negative	Positive	33.18
4	Positive	Positive	26.15	44	Negative	Negative	-	84	Positive	Positive	30.89	124	Positive	Positive	33.11
5	Positive	Positive	28.39	45	Negative	Negative	-	85	Positive	Positive	30.61	125	Negative	Negative	-
6	Negative	Negative	-	46	Negative	Negative	-	86	Positive	Positive	31.39	126	Positive	Negative	-
7	Positive	Positive	26.74	47	Negative	Negative	-	87	Positive	Positive	31.33	127	Negative	Negative	-
8	Negative	Negative	-	48	Negative	Negative	-	88	Negative	Positive	31.57	128	Negative	Negative	-
9	Negative	Negative	-	49	Negative	Negative	-	89	Negative	Positive	31.07	129	Negative	Positive	36.53
10	Positive	Positive	27.36	50	Negative	Negative	-	90	Negative	Positive	31.32	130	Negative	Negative	-
11	Negative	Negative	-	51	Negative	Negative	-	91	Negative	Positive	30.74	131	Negative	Negative	-
12	Positive	Positive	26.97	52	Negative	Negative	-	92	Negative	Positive	32.43	132	Negative	Positive	37.37
13	Positive	Positive	23.73	53	Negative	Negative	-	93	Negative	Positive	32.63	133	Positive	Positive	26.57
14	Positive	Positive	23.87	54	Negative	Negative	-	94	Negative	Positive	32.10	134	Negative	Negative	-
15	Positive	Positive	30.35	55	Negative	Negative	-	95	Negative	Positive	32.04				
16	Positive	Positive	20.76	56	Negative	Negative	-	96	Positive	Positive	27.17				
17	Negative	Negative	-	57	Negative	Negative	-	97	Negative	Positive	34.54				
18	Positive	Positive	31.84	58	Positive	Negative	-	98	Negative	Positive	32.94				
19	Negative	Positive	34.08	59	Positive	Negative	-	99	Negative	Positive	35.08				
20	Negative	Negative	-	60	Negative	Negative	-	100	Positive	Positive	27.49				
21	Negative	Positive	36.67	61	Negative	Negative	-	101	Negative	Positive	32.50				
22	Negative	Negative	-	62	Negative	Negative	-	102	Negative	Positive	34.51				
23	Negative	Positive	35.00	63	Negative	Negative	-	103	Negative	Positive	30.80				
24	Negative	Negative	-	64	Negative	Negative	-	104	Negative	Positive	31.45				
25	Negative	Negative	-	65	Negative	Negative	-	105	Positive	Positive	29.05				
26	Negative	Negative	-	66	Negative	Negative	-	106	Negative	Positive	39.45				
27	Negative	Positive	37.54	67	Negative	Negative	-	107	Negative	Positive	32.71				
28	Negative	Negative	-	68	Negative	Negative	-	108	Positive	Negative	-				
29	Negative	Negative	-	69	Negative	Negative	-	109	Positive	Positive	29.47				
30	Negative	Negative	-	70	Negative	Negative	-	110	Negative	Positive	32.67				
31	Positive	Positive	27.29	71	Negative	Negative	-	111	Negative	Positive	35.02				
32	Negative	Negative	-	72	Negative	Negative	-	112	Negative	Positive	34.66				
33	Negative	Negative	-	73	Positive	Positive	30.35	113	Positive	Positive	32.58				
34	Negative	Negative	-	74	Negative	Positive	38.85	114	Positive	Positive	30.45				
35	Negative	Negative	-	75	Negative	Negative	-	115	Negative	Positive	37.49				
36	Positive	Negative	-	76	Negative	Negative	-	116	Positive	Positive	34.27				
37	Positive	Negative	-	77	Negative	Positive	34.13	117	Negative	Positive	39.72				
38	Negative	Negative	-	78	Positive	Positive	25.26	118	Positive	Positive	28.79				
39	Negative	Negative	-	79	Positive	Positive	20.58	119	Negative	Positive	34.66				
40	Negative	Negative	-	80	Negative	Negative	-	120	Positive	Positive	32.72				

Ct=Cycle threshold, positive=Detected nucleic acid, negative=Undetected nucleic acid, qPCR=Quantitative polymerase chain reaction

**Table 3:** Performance of microscopy for *Ehrlichia* spp. detection compared with analysis of 134 whole blood dog samples examined in Nong Chock and Ladkrabang, Bangkok, Thailand.

Microscopy	qPCR		Performance

	Positive	Negative	
Positive	35	8	-
Negative	33	58	-
Sensitivity (%)	-	-	51.47
Specificity (%)	-	-	87.88
Accuracy (%)	-	-	69.40
Precision (%)	-	-	81.39

qPCR=Quantitative polymerase chain reaction

### Comparison of RHAM testing and qPCR for *Ehrlichia* spp. detection

The genomic detection of *Ehrlichia* spp. using the RHAM method was also assessed against qPCR, and the results are presented in Table 4. The RHAM test exhibited high diagnostic performance, with sensitivity, specificity, accuracy, and precision recorded at 91.18%, 98.48%, 94.78%, and 98.41%, respectively (Table 5).

**Table 4:** Results of genomic detection for canine Ehrlichiosis using RHAM technology, compared with quantitative qPCR.

No.	RHAM	qPCR		No.	RHAM	qPCR		No.	RHAM	qPCR		No.	RHAM	qPCR	
							
	Result	Result	Ct		Result	Result	Ct		Result	Result	Ct		Result	Result	Ct
1	Positive	Positive	32.57	41	Negative	Negative	-	81	Positive	Positive	29.83	121	Positive	Positive	33.30
2	Positive	Positive	23.49	42	Negative	Negative	-	82	Positive	Positive	30.24	122	Positive	Positive	31.99
3	Negative	Negative	-	43	Negative	Negative	-	83	Positive	Positive	29.96	123	Negative	Positive	33.18
4	Positive	Positive	26.15	44	Negative	Negative	-	84	Positive	Positive	30.89	124	Positive	Positive	33.11
5	Positive	Positive	28.39	45	Negative	Negative	-	85	Positive	Positive	30.61	125	Negative	Negative	-
6	Negative	Negative	-	46	Negative	Negative	-	86	Positive	Positive	31.39	126	Negative	Negative	-
7	Positive	Positive	26.74	47	Negative	Negative	-	87	Positive	Positive	31.33	127	Negative	Negative	-
8	Negative	Negative	-	48	Negative	Negative	-	88	Positive	Positive	31.57	128	Negative	Negative	-
9	Negative	Negative	-	49	Negative	Negative	-	89	Positive	Positive	31.07	129	Positive	Positive	36.53
10	Positive	Positive	27.36	50	Negative	Negative	-	90	Positive	Positive	31.32	130	Negative	Negative	-
11	Negative	Negative	-	51	Negative	Negative	-	91	Positive	Positive	30.74	131	Negative	Negative	-
12	Positive	Positive	26.97	52	Negative	Negative	-	92	Positive	Positive	32.43	132	Positive	Positive	37.37
13	Positive	Positive	23.73	53	Negative	Negative	-	93	Positive	Positive	32.63	133	Positive	Positive	26.57
14	Positive	Positive	23.87	54	Negative	Negative	-	94	Positive	Positive	32.10	134	Negative	Negative	-
15	Positive	Positive	30.35	55	Negative	Negative	-	95	Positive	Positive	32.04				
16	Positive	Positive	20.76	56	Negative	Negative	-	96	Positive	Positive	27.17				
17	Negative	Negative	-	57	Negative	Negative	-	97	Positive	Positive	34.54				
18	Positive	Positive	31.84	58	Negative	Negative	-	98	Positive	Positive	32.94				
19	Negative	Positive	34.08	59	Negative	Negative	-	99	Positive	Positive	35.08				
20	Negative	Negative	-	60	Negative	Negative	-	100	Positive	Positive	27.49				
21	Negative	Positive	36.67	61	Negative	Negative	-	101	Positive	Positive	32.50				
22	Negative	Negative	-	62	Negative	Negative	-	102	Positive	Positive	34.51				
23	Positive	Positive	35.00	63	Negative	Negative	-	103	Positive	Positive	30.80				
24	Negative	Negative	-	64	Negative	Negative	-	104	Positive	Positive	31.45				
25	Negative	Negative	-	65	Negative	Negative	-	105	Positive	Positive	29.05				
26	Negative	Negative	-	66	Negative	Negative	-	106	Positive	Positive	39.45				
27	Negative	Positive	37.54	67	Negative	Negative	-	107	Positive	Positive	32.71				
28	Negative	Negative	-	68	Negative	Negative	-	108	Positive	Negative	-				
29	Negative	Negative	-	69	Negative	Negative	-	109	Positive	Positive	29.47				
30	Negative	Negative	-	70	Negative	Negative	-	110	Positive	Positive	32.67				
31	Positive	Positive	27.29	71	Negative	Negative	-	111	Positive	Positive	35.02				
32	Negative	Negative	-	72	Negative	Negative	-	112	Positive	Positive	34.66				
33	Negative	Negative	-	73	Positive	Positive	30.35	113	Positive	Positive	32.58				
34	Negative	Negative	-	74	Negative	Positive	38.85	114	Positive	Positive	30.45				
35	Negative	Negative	-	75	Negative	Negative	-	115	Positive	Positive	37.49				
36	Negative	Negative	-	76	Negative	Negative	-	116	Positive	Positive	34.27				
37	Negative	Negative	-	77	Negative	Positive	34.13	117	Positive	Positive	39.72				
38	Negative	Negative	-	78	Positive	Positive	25.26	118	Positive	Positive	28.79				
39	Negative	Negative	-	79	Positive	Positive	20.58	119	Positive	Positive	34.66				
40	Negative	Negative	-	80	Negative	Negative	-	120	Positive	Positive	32.72				

Ct=Cycle threshold, positive=Detected nucleic acid, negative=Undetected nucleic acid, RHAM=RNase hybridization-assisted amplification, qPCR=Quantitative polymerase chain reaction

**Table 5:** Performance of genomic detection for canine Ehrlichiosis using RHAM Ehrlichiosis test kit, compared with qPCR.

RHAM test kit	qPCR		Performance

	Positive	Negative	
Positive	62	1	-
Negative	6	65	-
Sensitivity (%)	-	-	91.18
Specificity (%)	-	-	98.48
Accuracy (%)	-	-	94.78
Precision (%)	-	-	98.41

RHAM=RNase hybridization-assisted amplification, qPCR=Quantitative polymerase chain reaction

## DISCUSSION

Canine vector-borne diseases represent an escalating global concern due to their high morbidity and mortality rates in affected dog populations. Despite their clinical significance, data on their prevalence and distribution in Thailand remain limited. In the present study, 134 blood samples were collected from dogs exhibiting clinical signs suggestive of vector-borne infections in Bangkok during the 2024 rainy season. Microscopic examination revealed an overall apparent prevalence of 38.81% (52/134 dogs), with *Ehrlichia* spp. being the most commonly detected pathogen at 26.12%, followed by *H. canis* (6.72%), *Babesia* spp. (4.48%), *D. immitis* (3.73%), *Brugia* spp. (3.73%), and *Anaplasma* spp. (0.75%). Co-infections were identified in 5.22% of dogs (double infections) and 0.75% of dogs (triple infections). These findings align with the report of Rucksaken *et al*. [13], who documented a 36.73% prevalence of *Ehrlichia* spp. in northeastern Thailand using conventional PCR. Similarly, Vinnie-Siow *et al*. [21] reported a seroprevalence of 44.66% for *Ehrlichia* spp. in Malaysia. Conversely, Eamudomkarn *et al*. [22] identified *H. canis* as the predominant pathogen (65.71%) in brown dog ticks, followed by *Babesia* spp. (31.43%) and *Ehrlichia* spp. (30.00%), using conventional PCR. Díaz-Regañón *et al*. [23] also reported varying prevalence rates utilizing qPCR, including *H. canis* (31.43%), *A. platys* (31.43%), *E. canis* (27.14%), *Leishmania donovani* species complex (18.57%), *Theileria* spp. (12.86%), *Babesia vogeli* (12.86%), and *Babesia gibsoni* (2.86%).

This study also compared the diagnostic performance of routine microscopy against qPCR for the detection of *Ehrlichia* spp. Microscopic evaluation of Giemsa-stained blood smears remains widely utilized; however, its limitations are well-documented, particularly its dependence on elevated parasitemia levels and the necessity for skilled interpretation [24, 25]. Detection of *Ehrlichia* morulae in peripheral blood is infrequent, occurring in only 4%–6% of infected dogs. Sensitivity can be improved using buffy coat smears [26, 27], and expert cytologists may achieve up to 50% detection accuracy by examining lymph node aspirates [28]. Definitive diagnosis of acute canine Ehrlichiosis requires identification of *E. canis* morulae in mononuclear leukocytes from the buffy coat, spleen, cerebrospinal fluid, or bone marrow [29, 30]. Harrus *et al*. [31] reported morulae detection rates of 61%, 66%, and 74% when examining lymph nodes, buffy coat samples, and combined approaches, respectively. In this study, *Ehrlichia* spp. morulae were detected by microscopy in 26.12% of cases; however, eight false-positive and 33 false-negative results were observed when compared with qPCR. Differentiating morulae from artifacts and extraneous tissue structures remains a diagnostic challenge [32]. Notably, all false-negative cases corresponded to samples with Ct values exceeding 30.00, suggesting very low pathogen loads that hindered reliable microscopic identification.

Aziz *et al*. [19] emphasized the pivotal role of PCR for the accurate detection of canine Ehrlichiosis, highlighting its high analytical sensitivity capable of identifying minimal quantities of pathogen DNA. PCR not only allows differentiation between active and past infections but also facilitates early-stage diagnosis, which may be missed by serological methods. qPCR further refines diagnostic capabilities by enabling pathogen quantification. Multiplex and nested PCR approaches enhance the detection of co-infecting organisms [33–35], thereby broadening the diagnostic utility. Moreover, PCR is instrumental in evaluating persistent infections and treatment efficacy [36]. It is noteworthy that short-term doxycycline treatments may fail to eliminate *E. canis*, resulting in asymptomatic carrier states [37, 38]. Therefore, achieving a post-treatment PCR-negative status is recommended over relying solely on seronegativity [39]. Despite its diagnostic superiority, the widespread use of PCR remains limited by factors such as cost, infrastructure requirements, and turnaround time.

A tertiary objective of this study was to assess the diagnostic performance of a novel RHAM-based nucleic acid amplification platform. The RHAM technology combines loop-mediated isothermal amplification with RNase HII-mediated probe cleavage for fluorescence-based detection of specific target sequences. Amplification is facilitated by Bst DNA polymerase, while RNase HII cleaves the ribonucleotide within the DNA-probe duplex, releasing a fluorescence signal detectable within 30 min. An internal control incorporated into the assay monitors sample handling, nucleic acid release, and amplification efficiency, thereby minimizing false-negative and false-positive results. In this study, the RHAM Ehrlichiosis test kit (GuangzhouPluslife Biotech) demonstrated excellent performance compared with qPCR, achieving a sensitivity of 91.18%, specificity of 98.48%, accuracy of 94.78%, and precision of 98.41%. Nevertheless, six false-negative results were identified, all associated with Ct values >33.00, indicating that RHAM may have reduced sensitivity in cases with very low pathogen titers. Furthermore, the volume of blood used for RHAM testing (50 μL) was substantially lower than that used for qPCR, potentially influencing sensitivity. A single false-positive result may have been attributable to cross-contamination during sample processing.

Collectively, these findings highlight the RHAM test kit as a rapid, reliable, and practical diagnostic tool for the detection of *Ehrlichia* spp., particularly in decentralized or resource-constrained veterinary settings. To the best of our knowledge, this study represents the first field validation of the RHAM Ehrlichiosis test kit (GuangzhouPluslife Biotech), commercially launched in mid-2024, using qPCR as the gold standard comparator in a naturally infected canine population.

## CONCLUSION

This study investigated the prevalence of canine vector-borne pathogens and evaluated the diagnostic performance of microscopy, RHAM technology, and qPCR for detecting *Ehrlichia* spp. in clinically suspected dogs in Bangkok, Thailand. Microscopy demonstrated limited sensitivity and considerable false-negative rates, particularly in samples with low parasitemia, whereas qPCR confirmed its superior diagnostic accuracy. The novel RHAM Ehrlichiosis test kit (GuangzhouPluslife Biotech) exhibited high sensitivity, specificity, accuracy, and precision, approaching the diagnostic performance of qPCR while offering rapid, practical, and field-deployable testing capabilities.

The major strength of this study lies in its direct field validation of RHAM technology in a naturally infected canine population, providing real-world applicability data following its recent market release in 2024. Moreover, the study employed comparative evaluation against the current gold-standard qPCR, ensuring robust methodological rigor. However, several limitations should be acknowledged. First, the study population consisted solely of symptomatic dogs presenting at veterinary clinics, which may limit extrapolation to asymptomatic or broader canine populations. Second, although RHAM demonstrated excellent diagnostic performance, its sensitivity decreased in samples with extremely low pathogen titers, as reflected by qPCR Ct values greater than 33.00. Finally, the relatively small volume of blood used in RHAM testing compared to qPCR may have influenced detection rates.

The RHAM test kit represents a promising diagnostic alternative for the detection of canine Ehrlichiosis in resource-limited and decentralized settings, offering significant advantages in speed, ease of use, and diagnostic accuracy. Further large-scale, multi-Center studies including asymptomatic and subclinical cases are warranted to confirm its broader clinical utility and optimize diagnostic algorithms for canine vector-borne diseases.

## AUTHORS’ CONTRIBUTIONS

WP, TS, ThiS, RL, TT, and SR: Conceptualization. WP, TS, and SR: Formal analysis. SR: Investigation. WP, TS, RL, TT, and SR: Methodology. WP, TS, and SR: Project administration. SR: Supervision. ThiS and SR: Writing – original draft. WP, TS, and SR: Writing – review and editing. All authors have read and approved the final manuscript.
